# Waist circumference cutoff identifying risks of obesity, metabolic syndrome, and cardiovascular disease in men with spinal cord injury

**DOI:** 10.1371/journal.pone.0236752

**Published:** 2020-07-29

**Authors:** Satinder Gill, Ryan M. Sumrell, Adam Sima, David X. Cifu, Ashraf S. Gorgey

**Affiliations:** 1 Spinal Cord Injury and Disorders, Hunter Holmes McGuire VA Medical Center, Richmond, VA, United States of America; 2 Department of Biostatistics, Virginia Commonwealth University, Richmond, VA, United States of America; 3 Physical Medicine and Rehabilitation, Virginia Commonwealth University, Richmond, VA, United States of America; University of Perugia, ITALY

## Abstract

**Objectives:**

To apply spinal cord injury (SCI) specific waist circumference (WC) cutoff point to identify risks of 1) obesity, 2) metabolic syndrome (MetS), 3) cardiovascular disease (CVD).

**Methods:**

Thirty-six men with chronic SCI underwent anthropometric measurements, dual-energy x-ray absorptiometry (DXA), and magnetic resonance imaging (MRI) to measure total and regional adiposity. An SCI specific WC cutoff point of 86.5 cm was applied to the existing general population criteria. Pearson chi-square (χ^2^) analyses tested the difference in the number of participants classified as obese using the SCI specific cutoff point compared to the general population criteria. Sensitivity and specificity analyses relative to percentage body fat mass and visceral adipose tissue was used to assess classification performance of this cutoff point. The interrater reliability for three definitions of MetS was assessed using Cohen’s Kappa (*κ*) values. Linear regression analyses were utilized to propose SCI specific Framingham Coronary Heart Disease Risk Score (FRS) cutoff value.

**Results:**

Using SCI specific WC cutoff point of 86.5 cm, 36% of participants were classified as obese compared to only 3% when using WC of 102 cm *(P* < 0.001). Relative to percentage body fat mass, the general population WC cutoff point of 102 cm had a sensitivity of 6.3% and specificity of 100% both which changed to 68.8% and 90%, respectively, with a SCI specific cutoff point of 86.5 cm. Similar results were obtained when using visceral adipose tissue as a reference. The Kappa (*κ*) values improved substantially after using SCI specific criteria (0.95 ± 0.05) compared to the general population criteria (0.47 ± 0.28) for three definitions of MetS. The SCI specific FRS cutoff value of 6 was predicted after applying a WC cutoff of 86.5 cm.

**Conclusions:**

Using the existing general population criteria underestimated persons with SCI who are at risk of developing obesity, MetS, and CVD. The recommended SCI specific criteria are likely to distinguish those at risks of developing comorbidities and allow healthcare providers to intervene in a timely manner.

## Introduction

Persons with spinal cord injury (SCI) are at heightened risks of developing obesity, metabolic syndrome (MetS), and cardiovascular disease (CVD) [1,2]. Two out of three persons with SCI are considered obese and 55–57% of the entire population of individuals with SCI suffers from MetS [2,3]. Current literature suggests that the cluster of risk factors associated with MetS remains unclear; however, insulin resistance and central obesity have been major components of the syndrome [4]. The cumulative effects of obesity and MetS exacerbate the risk for CVD after SCI and it has been approximated at 228% that of able-bodied controls [5,6]. Obesity, MetS, and CVD have been attributed to decreased levels of physical activity, dysfunction in the autonomic nervous system, and changes in body composition [1]. Moreover, reduced physical activity levels combined with aging can lead to sarcopenic obesity. It has been shown that sarcopenia was more prevalent in persons with SCI when compared to age-matched able bodied participants [1,7,8]. This cluster of comorbidities contributes to reduced quality of life, increased economic burden, and mortality among persons with SCI.

Previous trials have relied on commonly available indices, such as waist circumference (WC) and body mass index (BMI) to identify the risk of obesity in persons with SCI [8–10]. Furthermore, others have used definitions drafted by the National Cholesterol Education Project Adult Treatment Panel III (NCEP ATP III), American Heart Association (AHA), and the International Diabetes Federation (IDF) to identify risks of MetS in persons with SCI. Framingham Coronary Heart Disease Risk Score (FRS) has also been used to assist providers in clinical determination of CVD risk prediction scores after SCI. The FRS is a multivariable model derived from studies in able-bodied population that intended to identify 10-year CVD risk factors. It is a sex-specific scoring system in which risk assessment is based on the individual’s total cholesterol (TC), high-density lipoprotein-cholesterol (HDL-C), age, sex, smoking status, level of blood pressure, diagnosis with diabetes and current treatment status.

At present, there are no SCI specific criteria that can provide a risk assessment of obesity, MetS, and CVD in SCI population. Reliance on the existing able-bodied indices and criteria likely underestimates the magnitude of the prevalence of this cluster in the SCI population. To address this limitation, a Canadian group has recommended using a BMI of > 22 kg/m^2^ to identify the risk of obesity in persons with SCI [8]. The general population BMI criteria has been shown to underestimate percentage body fat mass (FM) after SCI [1,2,8,9]. This modification has been considered by other groups who were interested in studying prevalence of MetS in men with SCI; [3] however, BMI is used only as a surrogate of whole-body adiposity and the index does not clearly account for central adiposity.

Central adiposity is characterized by increased visceral adipose tissue (VAT) in both able-bodied and persons with SCI. Persons with SCI have 58% greater central adiposity compared to the healthy controls, [10] which is tightly associated with abnormal metabolic profile [11]. Recent findings suggested that VAT negatively impacts the metabolic profile via secretion of pro-inflammatory cytokines in person with SCI [12]. Furthermore, VAT has been shown to account for lower level of cumulating testosterone in men with SCI [13]. The findings indirectly suggested the influence of VAT on pituitary-hypothalamic-testicular axis responsible for circulating testosterone and maintenance of lean mass in persons with SCI. Ryan et al. clearly showed that after dichotomizing based on VAT cross-sectional area (CSA) > 100 cm^2^ into two groups, peak oxygen uptake, triglycerides, insulin sensitivity, and glycated hemoglobin were different between groups [14]. Considering the limited accessibility of measuring VAT, the authors recommended using supine WC which was shown to be significantly associated with VAT CSA. Supine WC was chosen because it is less dependent on abdominal muscle tone, which is impaired in persons with SCI. Using a linear regression model the authors used a supine WC of 86.5 cm as SCI specific cutoff point to identify those who are at risk of developing central adiposity and associated metabolic derangement in lipid and carbohydrate profiles [14]. The findings further indicated that WC cutoff point of 86.5 cm may distinguish between those with higher TG and those with lower insulin sensitivity.

Since previous work did not account for central adiposity, we proposed applying the 86.5 cm SCI specific WC cutoff point to modify the existing criteria. This would provide clinicians with an accessible assessment tool to accurately distinguish risks of developing obesity, MetS, and CVD in the SCI population. The purpose of this study was to use a WC cutoff point of 86.5 cm to modify the existing classification criteria for the aforementioned comorbidities. Additionally, this WC cutoff point was applied to the definition of the MetS classification that were set by the three organizations (NCEP ATP III, AHA, and IDF), to develop modified SCI specific criteria for MetS. Finally, SCI specific FRS value was predicted to propose new cutoff value for CVD risk assessment in this population.

## Materials and methods

### Participants

Thirty-six men aged between 18–61 years, with chronic SCI (C5-L1; American Spinal Cord Injury Classification A-C) participated in one of the two clinical trials (registered at clinicaltrials.gov: NCT01652040 and NCT02660073). Participants were excluded if they had any pre-existing medical conditions that may have complicated their participation in study-specific exercises, such as coronary heart disease or current stage 2 pressure ulcers. Each protocol was approved by Institutional Review Boards at Hunter Holmes McGuire VA Medical Center and Virginia Commonwealth University. Following written consent, a physical examination was performed by a certified physician for each participant.

### Anthropometric measurements

Body weight was determined by subtracting the weight of each participant’s wheelchair from the weight of both the participant and wheelchair to the nearest 0.1 kg (Tanita, PW-63OU). Participants were positioned in a supine position to measure their height using a stadiometer (within 0.1 cm). BMI was calculated from these measures ($BMI={Weight}_{kg}/{\left({Height}_{m}\right)}^{2}$). WC was measured at the midpoint between the iliac crests and the inferior margin of the last rib while in a supine position using a standard inflexible measuring tape (MFG, Lufkin, Executive Diameter Pocket Tape Measure). The supine position was selected to mimic participant’s position at the MRI and DXA scanners. Hip circumference was also measured as the largest distance between the subject’s two greater trochanters. Each of these measurements was averaged using 3 trials (within 0.5 cm) and rounded to the nearest 0.1 cm. An SCI specific waist to hip ratio of 0.87 was used which is equivalent to 86.5 cm.

### Body composition

Whole body composition measurements such as total body fat mass (FM; kg) and total body fat percentage were measured using dual-energy x-ray absorptiometry (DXA). Both Lunar Prodigy and iLunar DXA scanners (GE Medical systems, Madison, WA) were used for body composition assessment. The precision of using DXA to measure total and regional body composition has been determined in persons with SCI [15]. Participants were instructed to remove all accessories and all measurements were performed after assuming a supine position for 20 minutes. Percentage body FM of 30% was used as a criterion value to ensure the accuracy of the applied SCI specific WC cutoff value of 86.5 cm. Recent Paralyzed Veterans of America (PVA) guidelines assumed definitions of obesity as percentage body FM greater than 22% [16]. We have chosen this cutoff based on previous work that clearly indicated that cardiometabolic dysfunction is likely to occur at percentage body FM of 30% in persons with chronic SCI [2,9,11].

### Magnetic resonance imaging

A General Electric Signa 1.5-Tesla magnet (Waukesha, WI) whole body scanner, using fast spin-echo sequence, was used to capture abdominal images [17]. Transverse images (slice thickness: 0.8 cm and inter-slice spacing: 1.2 cm) were acquired from the xiphoid process to the femoral heads. Depending on the length of participant’s torso approximately 20–30 images were captured. These images were analyzed using Win Vessels software (Ronald Meyer, Michigan State University, East Lansing, MI). Each of these images were automatically separated into fat and muscle, with bone and back-ground tissue identified based on its signal intensity [11–14,18,19]. An experienced researcher manually highlighted regions of interest to separate adipose tissue into VAT and subcutaneous adipose tissue (SAT). Total trunk CSA was defined as the total area within the outer border of the trunk.

Six participants were not able to participate in MRI due to presence of metallic implants in their bodies. For these participants trunk VAT CSA was estimated using Eq 1 [18] where DXA-VAT_VOL_ is the android region VAT volume measured using DXA and MRI-VAT_CSA_ is used to estimate VAT CSA.

$$MRI\;VAT_{CSA}=\left(DXA-VAT_{VOL}-123.18\right)/9.0318$$ (1)

### Lipid panel and intravenous glucose tolerance test

After overnight fasting for 10–12 hours, blood samples were drawn at the clinical research center. An intravenous cannula was inserted into the antecubital vein to draw blood samples into serum separator and potassium oxalate/sodium fluoride tubes. Serum and plasma were separated using centrifugation immediately at 3°C and 3000 rpm for 10 min. High-density lipoprotein cholesterol (HDL-C), low-density lipoprotein cholesterol (LDL-C), total cholesterol (TC) and triglycerides (TG) were analyzed using enzymatic colorimetric quantification.

Intravenous glucose tolerance test (IVGTT) was carried out by inserting an additional intravenous cannula into the opposite arm. Participants were administered glucose (0.3g/kg over 30 seconds) followed by a bolus of insulin (0.02U/kg) twenty minutes later. Blood samples were drawn from the opposite arm every 1–3 minutes (first 30 minutes), 5–10 minutes (next 40 minutes) and every 20 minutes (last 80 minutes). Plasma insulin was measured by ELISA (ALPACO, Salem, NH) and glucose was measured using a glucose analyzer.

### Obesity classification

Obesity was determined using WC, both general population and SCI specific cutoff points were used. The WC SCI specific cutoff point of > 86.5 cm [14] was used and replaced the existing general population cutoff point of ≥ 102 cm [20]. Classification accuracy of these WC cutoff points was compared against percentage body FM of 30% (“obese”: percentage body FM > 30% and “not obese”: percentage body FM < = 30%) and VAT CSA (VAT_CSA_) of 100 cm^2^ (“centrally obese”: VAT_CSA_ > = 100 cm^2^ and “not centrally obese”: VAT_CSA_ < 100 cm^2^).

### MetS classification

Metabolic syndrome classification was compared using a previously published general population criteria from three different organizations. The third National Cholesterol Education Program Adult treatment (NCEP ATP III) [21], the American Health Association (AHA) [22], and International Diabetes Federation (IDF) [23] were chosen. Participants were dichotomized into either “risk” or “no risk” classes for MetS. General population criteria for each of these organizations are presented in Table 1A. The SCI specific criteria for each definition was modified by using SCI specific WC cutoff point of 86.5 cm as listed in Table 1B.

**Table 1:** Metabolic syndrome using three different definitions that apply waist circumference as primary criteria.

**(a): General population criteria**	
National Cholesterol Education Program Adult Treatment Panel III (NCEP ATP III)	Presence of 3 or more of the following:
	• WC ≥ 102 cm • TG ≥ 150 mg/dL • HDL-C < 40 mg/dL • SBP ≥ 130 mm Hg or DBP ≥ 85 mm Hg • FBG ≥ 110 mg/dL
American Heart Association (AHA)	Presence of 3 out of the 5 following:
	• WC ≥ 102 cm • TG ≥ 150 mg/dL • HDL-C < 40 mg/dL • SBP ≥ 130 mm Hg or DBP ≥ 85 mm Hg • FBG ≥ 100 mg/dL or on specific treatment
International Diabetes Federation (IDF)	WC ≥ 94 cm or BMI > 30 kg/m^2^
	Plus any two of following:
	• TG ≥ 150 mg/dL • HDL-C < 40 mg/dL • SBP ≥ 130 mm Hg or DBP ≥ 85 mm Hg • FBG ≥ 100 mg/dL
**(b): SCI specific criteria**	
National Cholesterol Education Program Adult Treatment Panel III (NCEP ATP III)	Presence of 3 or more of the following:
	• WC > 86.5 cm • TG ≥ 150 mg/dL • HDL-C < 40 mg/dL • SBP ≥ 130 mm Hg or DBP ≥ 85 mm Hg • FBG ≥ 110 mg/dL
American Heart Association (AHA)	Presence of 3 out of the 5 following:
	• WC > 86.5 cm • TG ≥ 150 mg/dL • HDL-C < 40 mg/dL • SBP ≥ 130 mm Hg or DBP ≥ 85 mm Hg • FBG ≥ 100 mg/dL
International Diabetes Federation (IDF)	WC > 86.5
	Plus any two of following:
	• TG ≥ 150 mg/dL • HDL-C < 40 mg/dL • SBP ≥ 130 mm Hg or DBP ≥ 85 mm Hg • FBG ≥ 100 mg/dL

Body Mass Index: BMI; Waist Circumference: WC; Systolic Blood Pressure: SBP; Diastolic Blood Pressure: DBP; High Density Lipoprotein Cholesterol: HDL-C; Triglycerides: TG; Fasting Blood Glucose: FBG.

### Cardiovascular disease (CVD) risk score

We used the Framingham risk score (10-year) as a measure of the overall risk of CVD [24]. The following risk factors were incorporated: age, HDL-C, TC, systolic blood pressure (SBP), diagnosis with diabetes, smoking status, and blood pressure medication. A risk score of ≤ 10 is considered as “no risk” class, and > 10 as “risk” class. The SCI specific FRS cutoff value was calculated using WC SCI specific cutoff point of 86.5 cm. Participants having WC > 86.5 cm were classified at “risk” of CVD and those having WC ≤ 86.5 were classified as at “no risk”.

### Statistical analysis

Statistical analyses were performed using SAS^®^ (2019a, Cary, NC, United States). All data were tested for normality using Shapiro-Wilk test. The distributions of TG and fasting blood glucose (FBG) were positively skewed and were log-transformed. Linear regression analyses were used to examine the relationships between the WC and percentage body FM, VAT_CSA_, CVD risk factors, and the Framingham risk score. The concordance of the obesity indicators obtained from WC compared to WHR were assessed using the kappa statistic. Cohen’s Kappa (***κ***) values were interpreted in the context of Landis et al. (k < 0.00: poor, 0.00–0.20: slight, 0.21–0.40: fair, 0.41–0.60: moderate, 0.61–0.80: substantial, 0.81–1.00) [25]. Analysis of the sensitivity and specificity of general population and SCI specific WC cutoffs for obesity was computed relative to percentage body FM of 30% and VAT_CSA_ of 100 cm^2^.

Positive and negative predictive values (PPV, NPV) were calculated for each indicator’s ability to identify metabolic syndrome defined by each of the three previously discussed organizations. The NCEP and AHA definitions were confined since no differences were observed between these two definitions in our data. Receiver operating curves (ROC) were calculated to assess the discriminative relationship between each of the obesity outcomes and the CVD measure for total body fat and VAT.

Two-tailed independent t-tests were used to determine the significant differences (*P* < 0.05) between groups for TG, HDL-C, and FBG for three definitions of MetS. The statistical differences between FRS cutoff values of 10 and 6 for the CVD risk factors (Age, HDL-C, TC, and SBP) were calculated using two-tailed independent t-tests.

## Results

Participant demographics, injury characteristics, anthropometric measurements, and cardiometabolic profiles are presented in Table 2. Sixty-seven percent of the participants were classified as paraplegic and 33% were tetraplegic.

**Table 2:** Physical characteristics and metabolic profile of all participants enrolled in the current study.

	Total (n = 36)	Paraplegic (n = 24)	Tetraplegic (n = 12)	P value
**Demographics**				
Age (y)	37 ± 11	36 ± 11	37 ± 12	0.85
Smoking Status (y/n)	21/15	14/10	7/5	-
Diabetes	0	0	0	-
Diabetes Medication (y/n)	9/27	4/20	5/7	-
**Injury Characteristics**				
Neurological level of injury	C5 –L1	T4 –L1	C5 –C8	-
		T4 (n = 5), T5 (n = 3), T6 (n = 6), T8 (n = 4), T10 (n = 2), T11 (n = 2), T12 (n = 1), L1 (n = 1)	C5 (n = 2), C6 (n = 8), C7 (n = 1), C8 (n = 1)	

AIS classification	A–C	A–C	A–C	-
		A (n = 17), B (n = 6), C (n = 1)	A (n = 8), B (n = 3), C (n = 1)	
Time Since Injury (y)	9 ± 9	10 ± 10	9 ± 8	0.79
**Anthropometric Measurements**				
Body Mass (kg)	73.8 ± 13.9	75.1 ± 13.5	71.3 ± 15.0	0.48
BMI	23.4 ± 4.5	23.7 ± 4.1	22.7 ± 5.4	0.51
WC (cm)	82.1 ± 12.3	81.6 ± 11.5	83.1 ± 14.1	0.74
**Blood Pressure**				
SBP (mm/Hg)	115.1 ±19.6	119.5 ± 16.7	106.3 ± 22.7	0.06
DBP (mm/Hg)	71.2 ± 10.9	73.5 ± 8.9	66.5 ± 13.3	0.08
**Lipid Profile**				
LDL-C (mg/dL)	94 ± 25.2	93.1 ± 25.7	95.7 ± 25.1	0.77
HDL-C (mg/dL)	37.6 ± 8.9	38.8 ± 9.5	35.1 ± 7.4	0.25
TC (mg/dL)	153.1 ± 27.2	151.6 ± 27.1	156.0 ± 28.2	0.65
TG (mg/dL)	106.1 ± 52.8	97.9 ± 46.3	122.4 ± 63.1	0.20
**Carbohydrate Profile**				
Fasting Glucose ^a^	90.7 ± 13.9	89.9 ± 10.1	92.5 ± 20.6	0.63
Fasting insulin ^b^	3.4 ± 2.2	3.5 ± 2.5	3.4 ± 1.3	0.92

AIS-A: complete motor and sensory loss below the level of injury; AIS-B: complete motor and incomplete sensory loss below the level of injury; AIS-C: incomplete motor and sensory loss below the level of injury with less than 50% of motor sparing; AIS-D: incomplete motor and sensory loss below the level of injury with more than 50% of motor sparing); Body Mass Index: BMI; Waist Circumference: WC; Systolic Blood Pressure: SBP; Diastolic Blood Pressure: DBP; Low Density Lipoprotein Cholesterol: LDL-C; High Density Lipoprotein Cholesterol: HDL-C; Total Cholesterol: TC; Triglycerides: TG; ^a^n = 35; ^b^n = 34; P: significance value for independent t-test used to determine statistical differences between Paraplegic and Tetraplegic groups for age, time since injury, body mass, BMI, WC, SBP, DBP, LDL-C, HDL-C, TC, TG, fasting glucose and fasting insulin.

### Obesity

**Fig 1:**
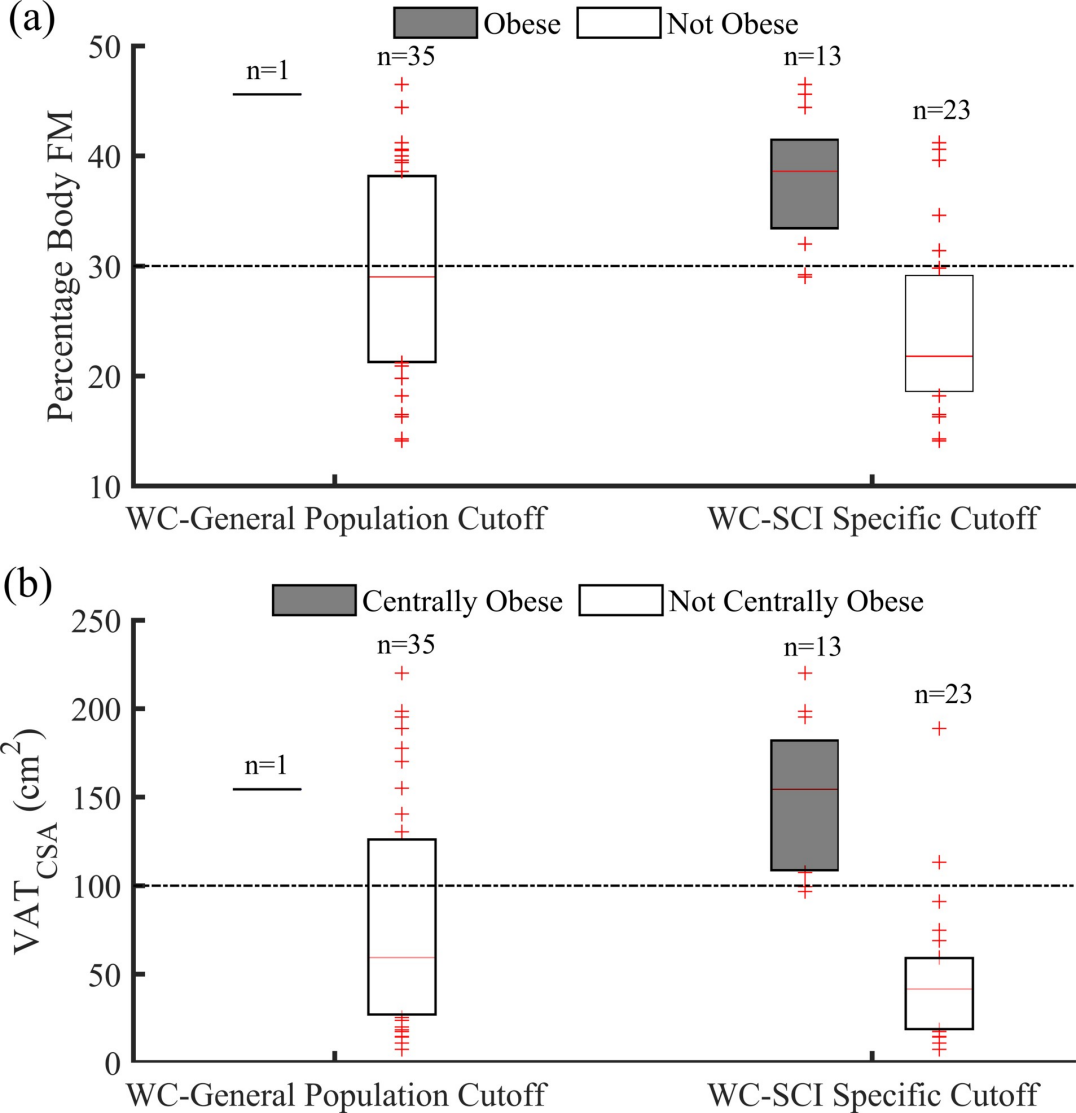
(a): Distribution of percentage body fat mass for participants classified as “obese” and “not obese” using general population (102 cm) and SCI specific (86.5 cm) cutoff points for WC. (b): Distribution of visceral adipose tissue cross-sectional area for participants classified as “centrally obese” and “not centrally obese” using general population (102 cm) and SCI specific (86.5 cm) cutoff points for WC. Data are presented using box plots. The optimal cutoff points (percentage body fat mass of 30% and visceral adipose tissue cross-sectional area of 100 cm^2^) are represented using black dotted lines.

Almost two-thirds of the sample (n = 13, 36%) were identified as obese when using the SCI specific WC cut-off. This number was similar to those considered obese using the WHR cut-off ratio of 0.87 (n = 16, 44%). The WC was positively related with both percentage body FM (r^2^ = 0.68, *P* < 0.001) and VAT_CSA_ (r^2^ = 0.64, *P* < 0.001). Fig 1A shows the distribution of percentage body FM for those classified as “obese” and “not obese” using general population and SCI specific WC cutoff points. Similarly, Fig 1B shows the distribution of VAT_CSA_ for those classified as “centrally obese” and “not centrally obese” using these cutoff points for WC. There is a clear separation between percentage body FM for those classified as “obese” and “not obese” using SCI specific WC cutoff point. Similarly, VAT_CSA_ for those classified as “centrally obese” and “not centrally obese” was clearly separated when SCI specific WC cutoff point of 86.5 cm was used.

The WC cut-off for obesity aligned with the WHR cut-offs in 81% (29/36) of the participants. Kappa values for these comparisons were 0.60 (95% CI: 0.34, 0.86; *P* = 0.25). When comparing the WC and WHR indications of obesity, all 9 discordant participants were indicated as obese using the WHR threshold but not obese using the WC threshold.

The area under the curve (AUC) for WC relative to percentage body FM and VAT_CSA_ was 0.88 (for both). Relative to percentage body FM, the general population WC cutoff point of 102 cm had a sensitivity of 6.3% and specificity of 100% both which changed to 68.8% and 90%, respectively, with a SCI specific cutoff point of 86.5 cm (Table 3). In reference to VAT CSA, the general population WC cutoff point had a sensitivity of 7.7% and specificity of 100% both of which changed to 84.6% and 91.3% respectively, with a SCI specific cutoff point of 86.5 cm (Table 3).

**Table 3:** Comparison of sensitivity, specificity, predictive and prevalence values for comparison of obesity using WC cutoff values (102 cm vs 86.cm) with respect to percentage body fat mass and visceral adipose tissue CSA.

	Percentage body fat mass			Visceral adipose tissue CSA		
	Obesity WC Cutoff		% Change	Obesity WC Cutoff		% Change
	102 cm	86.5 cm		102 cm	86.5 cm	
**Sensitivity**	6.3%	68.8%	62.5%	7.7%	84.6%	76.9%
**Specificity**	100%	90%	-10%	100%	91.3%	-8.7%
**Predictive value**	58.3%	80.6%	22.3%	66.7%	88.9%	22.2%
**Positive predictive value**	100%	84.6%	-15.4%	100%	84.6%	-15.4%
**Negative predictive value**	57.1%	78.3%	21.2%	65.7%	91.3%	25.6%
**Prevalence**	44.4%	44.4%	0%	36.1%	36.1%	0%

True positive: TP; True negative: TN; False positive: FP; False negative: FN; Sensitivity = TP/(TP+FN); Specificity = TN/(FP+TN); Predictive value = (TP+TN)/(TP+FP+TN+FN); Positive predictive value = TP/(TP+FP); Negative predictive value = (TN/TN+FN); Prevalence = (TP+FN)/(TP+FP+TN+FN).

### MetS

**Fig 2:**
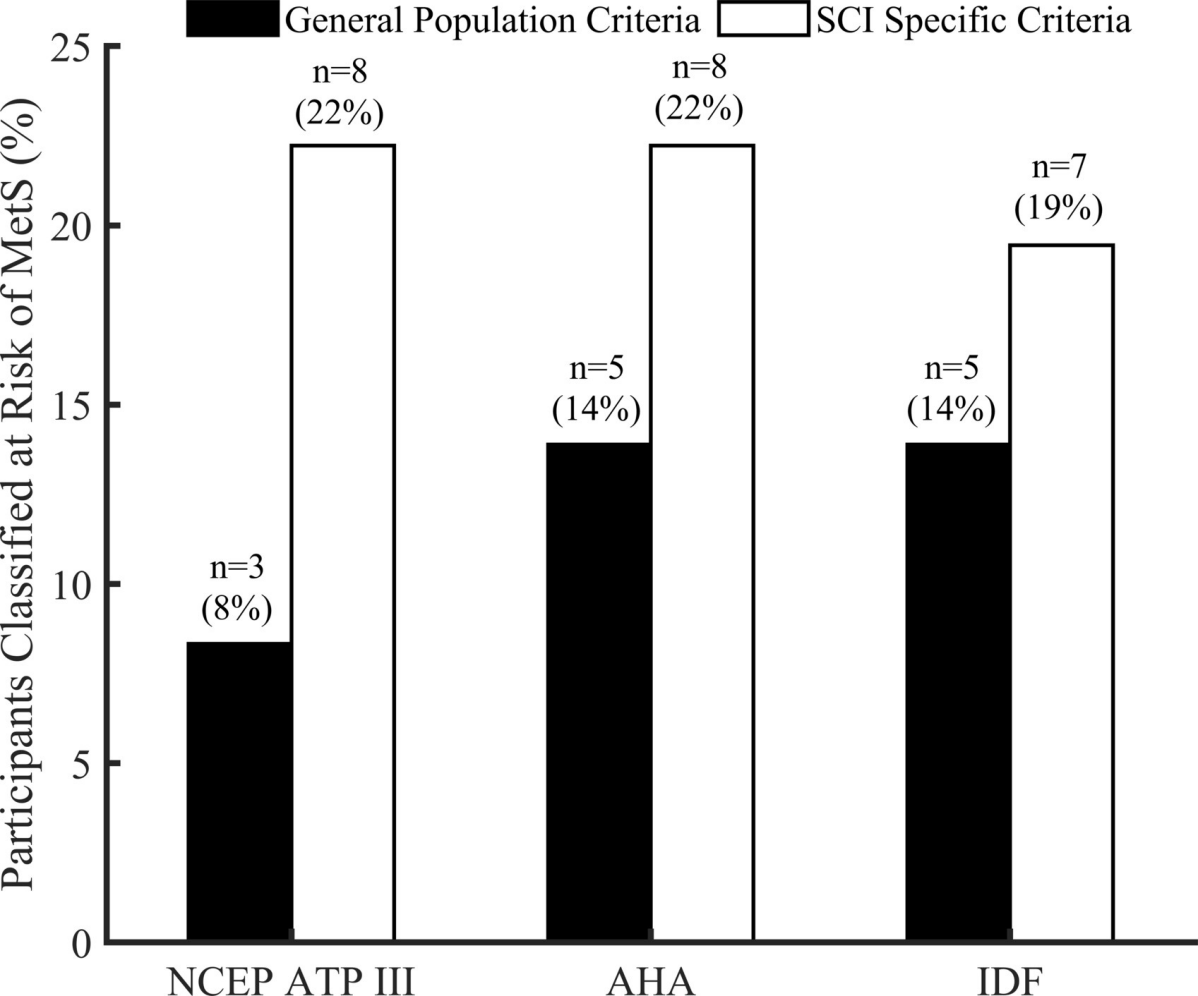
Prevalence of MetS for the three definitions using general population and SCI specific criteria listed in Table 1A and 1B respectively.

**Fig 3:**
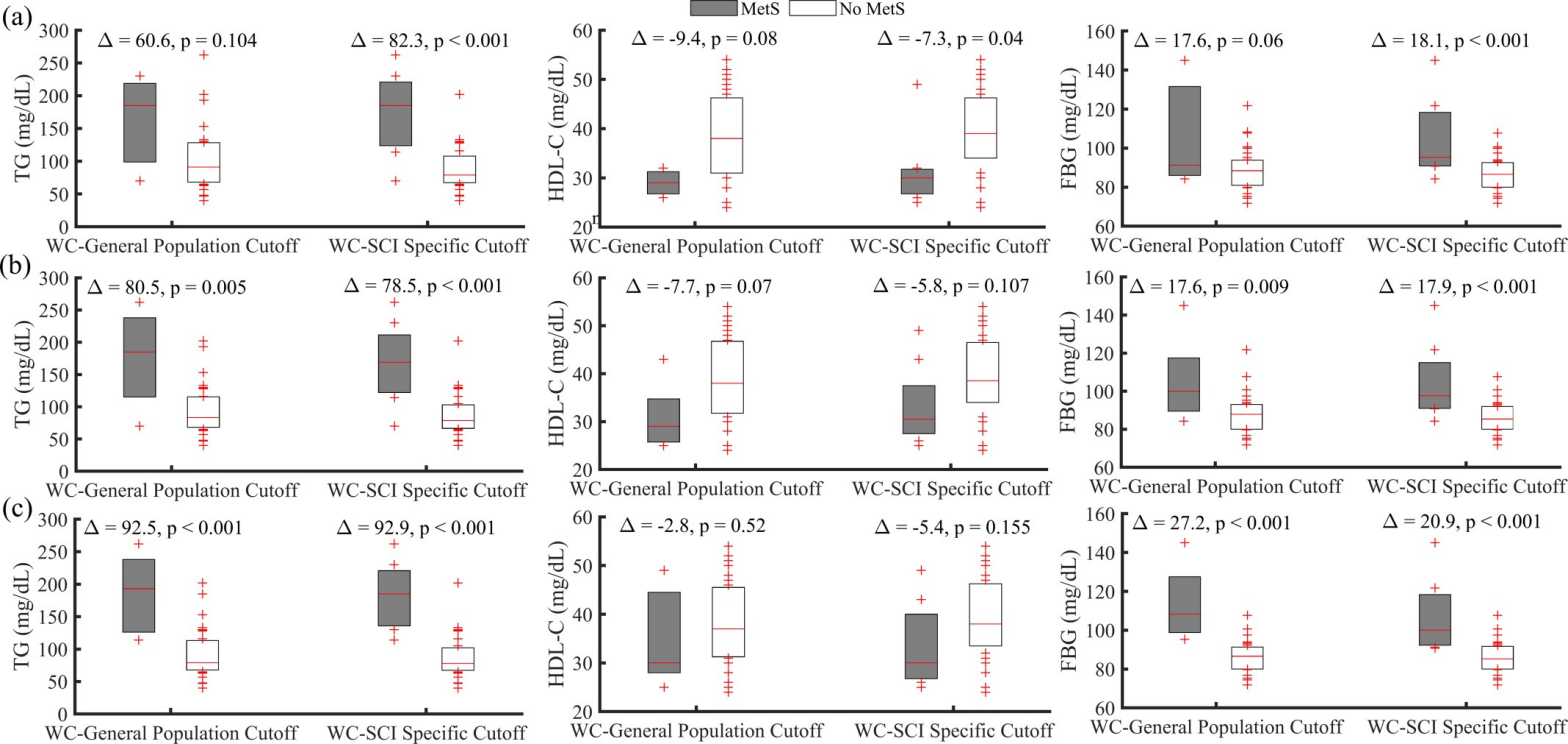
Distribution of triglycerides (TG), high density lipoprotein-cholesterol (HDL-C), and fasting blood glucose (FBG) level between those classified at risk “MetS” and “No MetS” using either general population (102 cm) or SCI specific (86.5 cm) cutoff points of WC. (a): NCEP ATP III, (b): AHA, and (c): IDF. Δ is the mean difference between these variables for participants classified at risk and no risk (“MetS” and “No MetS”). P is the significance value for independent t-test used to determine the differences between groups for TG, HDL-C, and FBG for three definitions of MetS.

As seen in Fig 2, using the general population criteria listed in Table 1A, the prevalence of MetS was 8% (NCEP), 14% (AHA), and 14% (IDF). When the SCI specific criteria, listed in Table 1B, was applied the prevalence of MetS was found to be 22%, 22% and 25% using the NCEP, AHA and IDF. Fig 3 shows distribution of TG, HDL-C and FBG for those classified at risk and no risk (“MetS” and “No MetS” respectively) using general population and SCI specific cutoff points of WC for NCEP (Fig 3A), AHA (Fig 3B) and IDF (Fig 3C). There is an indication that these variables are more separatable when SCI specific criteria of 86.5 cm was used for three MetS definitions. Specifically, statistically significant results were obtained for NCEP ATP III when SCI specific criteria was used.

Participants classified as obese on each of the SCI-specific indicators were excellent indicators of having metabolic syndrome, with PPVs ranging from 0.91 to 1.0. However, negative indicators of obesity were poor to fair in predicting the absence of metabolic syndrome, with NPVs ranging from 0.18 to 0.54. The WC cut-off nominally outperformed the WHR thresholds for NPV performance.

Lastly, the Kappa (*κ*) values indicating the interrater reliability for the three MetS definitions using general population criteria (0.47 ± 0.28) are presented in Table 4A. Similarly, interrater reliability values using SCI specific criteria (0.95 ± 0.05) for the three definitions are listed in Table 4B. The *κ* values listed in Table 4 indicated that the interrater reliability improved substantially when SCI specific criteria were used as compared to general population criteria.

**Table 4:** Cohen’s kappa (*κ*) agreement for three MetS definitions using different criteria.

**(a): General population criteria**			
	NCEP ATP III	AHA	IDF
NCEP ATP III	1	0.72	0.16
AHA	0.72	1	0.53
IDF	0.16	0.53	1
**(b): SCI specific criteria**			
	NCEP ATP III	AHA	IDF
NCEP ATP III	1	1	0.92
AHA	1	1	0.92
IDF	0.92	0.92	1

National Cholesterol Education Program Adult Treatment Panel III: NCEP ATP III; American Heart Association: AHA; International Diabetes federation: IDF

### Cardiovascular risk

WC was positively correlated with four CVD risk factors: age (r^2^ = 0.3, *P* < 0.001), TC (r^2^ = 0.14, *P* = 0.03), SBP (r^2^ = 0.11, *P* = 0.04), and smoking status (r^2^ = 0.10, *P* = 0.05). It was also positively correlated with the Framingham risk score (r^2^ = 0.12, *P* = 0.04).

**Fig 4:**
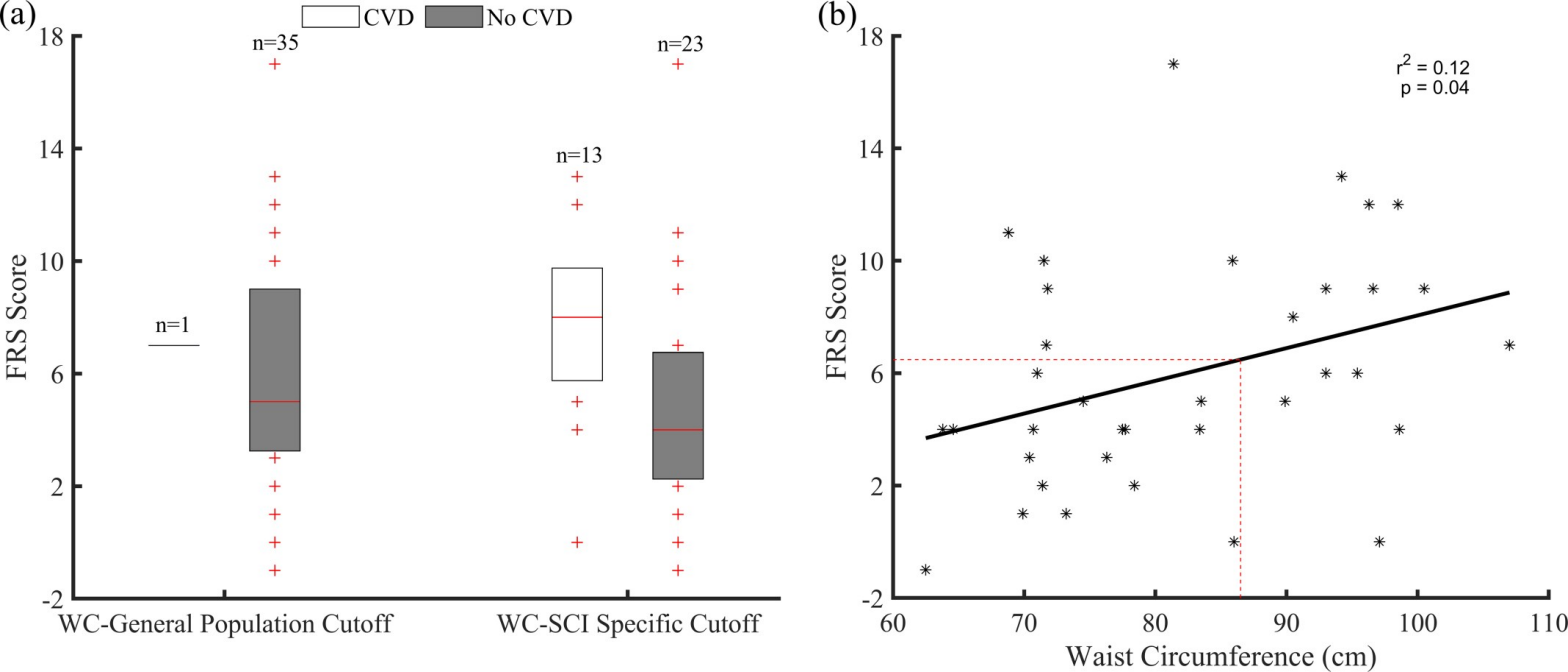
(a): Distribution of FRS score for those classified at risk “CVD” and “No CVD” using either general population (102 cm) or SCI specific (86.5 cm) cutoff points of WC. (b): Association between FRS score and waist circumference.

Fig 4A shows FRS distribution for those classified at “risk” and “no risk” using general population and SCI specific WC cutoff points. FRS score was separated when SCI specific cutoff of 86.5 cm was used when compared to general population cutoff point. Fig 4B shows the association between the FRS score and WC. Using the SCI specific WC cutoff point of 86.5 cm (dotted red line), the FRS value of 6 was obtained as an optimal cutoff for SCI population.

When using general population FRS cutoff value of 10 (risk: 9.4%), 14% (n = 5) of participants were classified at “risk” of CVD, while 86% (n = 31) were classified as “no risk”. When SCI specific FRS cutoff value of 6 (risk: 4.7%) was used, 36% (n = 13) of participants were classified at “risk”, while 64% (n = 23) were classified as “no risk”.

## Discussion

The major finding of this study was that applying SCI specific WC cutoff point (> 86.5 cm) improves the diagnostic ability of the existing indices and criteria to accurately capture those at risk of developing obesity, MetS, and CVD. Of note, the general population WC criteria underestimated the prevalence of obesity by 33% compared to the SCI specific cutoff point of 86.5 cm. On average, the risk of MetS increased by 1.5-fold after applying the SCI specific WC cutoff point. Furthermore, the separation in percentage body FM and VAT_CSA_ for those classified at risk of being “obese” and “not obese” increased when SCI specific WC cutoff point was applied. This provides credence to our hypothesis that central adiposity is the root of cardio-metabolic disorders in this population. Finally, an SCI specific FRS cutoff value of 6 was proposed to accurately predict those at risk of developing CVD.

The current work was based on previous work that demonstrated that VAT accumulation influences a number of metabolic parameters in persons with SCI, including glucose tolerance, lipid profile and circulating level of serum testosterone [11,13,14]. However, measurements of VAT were conducted primarily by expensive and inaccessible imaging techniques such as magnetic resonance imaging (MRI) and computed tomography (CT) [11,14,26]. To overcome such a barrier, we have developed a number of prediction equations that simply can use anthropometrics and DXA to accurately predict VAT [18,19]. We and others have previously shown that VAT CSA > 100 cm^2^ negatively impacts metabolic profile in persons with SCI [14, 26]. A Japanese group has clearly shown that VAT_CSA_ of 100 cm^2^ is equivalent to WC of 81 cm [26]. Ryan et al. noted that supine WC of 86.5 cm corresponds to VAT_CSA_ of 100 cm^2^ in people with chronic SCI [14]. The authors clearly demonstrated that at this point there is deviation in the lipid profile characterized by increased triglycerides, total cholesterol, and non-HDL-C as well as impaired insulin sensitivity in men with SCI [14].

Obesity is associated with diabetes, all-cause mortality, and CVD mortality after SCI [1–3]. In this study, the use of SCI specific WC cutoff point (> 86.5 cm) reflected both central adiposity and whole-body adiposity. A recent report suggested that in 155 veterans with SCI, 93% were considered at risk of developing at least one of the clusters of obesity, MetS and CVD [3]. The study clearly noted that there is no SCI WC cutoff point that has been validated for this population. For obesity risk, the study had used a WC cutoff of 94 cm and BMI of 22 kg/m^2^ to account for the existing general population WC and BMI cut-off points of 102 cm and 30 kg/m^2^, respectively. In previous report, using the SCI specific BMI cutoff point revealed 54% difference compared to the general population cutoff point and only 8% after using the SCI specific WC cut-off point [27]. In the current study, using WC of 86.5 cm showed 33% difference in the risk of obesity compared to the general population cutoff point of 102 cm. The discrepancy between the two studies may be attributed to the fact that their sample included both complete/incomplete SCI with American Spinal Injury Association (ASIA) classification Impairment Scale (AIS) A-D, whereas in the current study we primarily focused on AIS A and B.

For risk of MetS, previous studies have demonstrated great variability in the results after using the definitions set by different organizations. The number of participants classified with MetS were (NCEP (17%), AHA (53%), and IDF (31%)) [27]. The study conducted by Finnie *et al*. revealed a prevalence of NCEP (12.3%), AHA (15.8%), and IDF (19.3%) in a cohort of 56 people [28]. For IDF criteria, Gater et al. used SCI specific BMI cutoff point of (> 22 kg/m^2^) to reflect central obesity [3]. This was likely performed to account for lack of WC measurements among their cohort. However, a recent report clearly indicated that using BMI instead of WC is likely to underestimate the risk of MetS by 46% in this population [27]. Therefore, modifying the existing definitions may provide an early onset tool to capture MetS in this population. In the current study after using the SCI specific WC cutoff point (> 86.5 cm), the prevalence of MetS according to NCEP, AHA, and IDF was 22%, 22%, and 19%, respectively. Compared to the general population cutoff point, the risk of developing MetS was 1.5-fold greater than using NCEP, AHA, and IDF.

FRS was chosen because it is the most commonly used and recognized prediction score of CVD among clinicians [29]. There are some studies that have applied FRS in the SCI population, however, it has not been validated in a large cohort in this population [27]. The study conducted by Yahiro *et al*. revealed that 68% of veterans with SCI were considered at “risk” of CVD [27]. It has also been shown that WC was strongly correlated with many risk factors of FRS scale [30]. Based on these results, WC cutoff point of (≥ 94 cm) was proposed as an optimal cut-off point for CVD risk in participant’s with SCI [30]. However, application of traditional risk factors may not be appropriate in persons with SCI, mainly due to factors such as changes in body composition and impairment in autonomic nervous system with lesions above the sixth thoracic level. In the present study, only 14% of participants were classified at “risk” after using the general population FRS cutoff value of 10. Whereas, when proposed SCI specific FRS cutoff value of 6 was used 36% of participants were classified at “risk”. These results indicated that FRS score of 6 may facilitate early identification of CVD in this population. Although a lower FRS cutoff may better discriminate subjects for the presence of CVD risk factors, extreme caution should be considered because this was based on WC as a single surrogate in a small SCI population; which has the potential to lead to overdiagnosis and overtreatment.

### Limitations

There are considerable limitations of the present study that must be acknowledged. One of the major limitations is the small sample size. Unlike general population studies that are usually very large, the sample size is restricted by small population size. Furthermore, the use of advanced imaging technologies similar to DXA and MRI are costly and prohibitively impacted our ability to increase the sample size. In addition, these results may not accurately translate to women with SCI as only men participated in the current study. Further studies involving both men and women are warranted to ascertain the validity of SCI specific WC to detect the risk of obesity, MetS, and CVD across a larger cohort. Moreover, further prospective studies are warranted to apply the newly developed FRS cut-off point in clinical prediction of CVD after SCI. Lastly, the level of physical activity was not objectively measured in the current report. However, participants recruited in this study were primarily motor complete SCI and are therefore likely to have lowest level of physical activity.

## Conclusions

Application of traditional risk factor criteria underestimates the prevalence and magnitude of obesity, MetS, and CVD in persons with SCI. Developed SCI specific WC cut-off point resulted in the improved classification of those who are at the risk of obesity. Moreover, applying this WC cut-off point to definitions of MetS drafted by NCEP, AHA, and IDF improved their ability and precision to identify those at risk of MetS. Finally, the results suggest that there may be a greater difference in mean values of CVD risk factors for those classified at “risk” and “no risk” when the SCI-FRS cutoff value of 6 was used and this may enable early prediction of CVD in this population.
